# Training DAFNE *plus* facilitators in novel behaviour change approaches: A template for training design and delivery

**DOI:** 10.1111/dme.70078

**Published:** 2025-06-22

**Authors:** Nicole de Zoysa, Paul Chadwick, Carolin Ferguson, Carla Gianfrancesco, Julia Lawton, Simon Heller

**Affiliations:** ^1^ Diabetes Research King's College Hospital NHS Foundation Trust London UK; ^2^ Epidemiology and Public Health UCL London UK; ^3^ Diabetes Centre Sheffield Teaching Hospitals NHS Foundation Trust Sheffield UK; ^4^ Usher Institute University of Edinburgh Edinburgh UK; ^5^ Department of Oncology and Metabolism The University of Sheffield Sheffield UK

**Keywords:** action planning, behaviour change, DAFNE, DAFNE*plus*, diabetes educator, healthcare professional training, self‐compassion

## Abstract

**Background:**

The integration of behaviour change principles into diabetes structured education programmes is key to sustaining long‐term improvements in glycaemic management. Good quality training for diabetes educators that enables them to understand and feel confident in behaviour change approaches, is fundamental to their delivery. Educator training programmes are rarely described in detail despite this information being needed to enable interpretation of trial/outcome data, fidelity analyses and replicability between centres.

**Aims:**

This paper presents an overview of a bespoke training programme developed for educators who delivered the DAFNEplus programme. DAFNEplus is a revised version of the Dose Adjustment for Normal Eating Programme (DAFNE) for people with type 1 diabetes. The revisions incorporate novel technology, behaviour change approaches and structured individualised follow‐up.

**Materials and Methods:**

This paper outlines the structure of the training provided to educators, a summary of its development and examples of training content and process.

**Results:**

We provide a template for future training courses, where psychological approaches are integrated into standard diabetes education and delivered by healthcare professionals.

**Discussion:**

The impact upon educators' perceptions and delivery of structured education, as well as the potential for ongoing training development, is also discussed.

**What's New?**

- DAFNE*plus* is a revision of the Dose Adjustment for Normal Eating Programme (DAFNE) incorporating novel technology, behaviour change approaches and individualised follow‐up.
- A new educator training programme was required to cover these novel aspects and address gaps in the literature around initiating and sustaining behaviour change.
- Key processes around action planning, self‐compassion and educator communication were embedded in the programme, with the aim of supporting setback management and promoting long‐term resilience.
- This manuscript provides an overview of the training's structure, content and process and provides a template for future educator training where behaviour change approaches are integrated into structured education programmes.

## 1 DAFNE*plus* BACKGROUND

Optimal, sustained management of type 1 diabetes remains challenging despite high‐quality structured education programmes such as the Dose Adjustment for Normal Eating (DAFNE) programme.^1^ To address the challenges of long‐term maintenance, a revised programme called DAFNE*plus* was developed. DAFNE*plus* is a 1‐year programme comprising: a group course (once/week for 5 weeks),^2^ five individual follow‐up sessions, staggered over 1 year^3^ and a bespoke technology platform.^4^, ^5^ The DAFNE curriculum was revised through the lens of behaviour change science, specifically targeting the barriers and drivers to sustainable self‐management. This updated programme drew upon research highlighting the challenges DAFNE graduates experienced post‐course^6^, ^7^, ^8^ a behaviour change analysis of diabetes management,^9^, ^10^ a collaborative working group process to inform curriculum revisions^11^ and a programme theory to describe the potential outcomes and mechanisms of action.^12^ DAFNE*plus* is currently being evaluated within a randomised controlled trial.^13^

## 2 SUPPORTING WORKFORCE DEVELOPMENT

Recent recommendations to improve the quality of reporting in health psychology interventions suggest that the training for educators (usually nurses and dieticians) should be adequately described. This will support analysis of fidelity, interpretation of trial findings and enable replicability of the intervention outside the trial setting.^14^ Consequently, this paper focuses specifically upon the training developed for DAFNE*plus* educators (renamed Facilitators) to deliver this new programme.

The aim of the training programme was to upskill current DAFNE educators to deliver the novel DAFNE*plus* elements competently and confidently and address training gaps identified in the literature in initiating and sustaining behaviour change (as described below). A combination of literature review and collaborative group work led to the design of a bespoke training package that is described in this paper and may serve as a template for other health education programmes, with a behaviour change focus.

## 3 NOVEL DAFNE*plus* COMPONENTS

To understand the training requirements for the DAFNE*plus* trial, a brief summary of the novel elements of the programme is described below.

### 3.1 Clinical

The curriculum and timetable were changed so that sessions with a psychological focus could be included (e.g. managing emotions and mindset). Some of the more didactic content (e.g. what is diabetes?) was moved into an e‐learning format to make room for this.^9^ The timetable was changed to represent the three cycles of behavioural management, routine, reactive, reflective^10^ and accommodate layered learning (e.g. splitting hypoglycaemia teaching over several sessions). The curriculum's language was changed to reflect the recent ‘language matters’ guidance (e.g. ‘check’ instead of ‘test’ blood glucose)^15^ and support simplification of learning (e.g. single number targets instead of ranges), based on OzDAFNE health literacy revisions.^16^ Some clinical recommendations were also revised to reflect updated guidance, for example, aiming for 70% blood glucose within target range.

### 3.2 Psychological

Additional psychological content included new sessions addressing behaviour change principles (e.g. action planning and setback management). Psychological well‐being was prioritised within the structure of the programme, for example, asking about emotional reactions related to managing diabetes in the first group session and beginning each individual check‐in session by asking ‘what has gone well?’ first, before discussing problems. The previously entitled ‘annual review session’ was retitled ‘monitoring your long‐term health’ and re‐written from a behaviour change perspective to minimise fear and encourage engagement with screening for early prevention. New visual metaphors were introduced e.g. the messy cupboard (see Supplementary Material for more information) to describe the process of chaos to order when learning new ways to manage diabetes, and new materials were produced, such as an action planning workbook.

### 3.3 Technological

The technology changes included using a blood glucose meter with a bolus advisor feature and linking this to a data upload box that could be synched at home (Withcare+) and uploaded onto an online platform (the DAFNE*plus* website) where unique data displays could be seen such as the ‘dot chart’ which showed behaviours predicting hypoglycaemia recurrence. To encourage engagement with the new technology, gamification was used, for example, stars could be achieved for optimal data entry, and a ‘carb challenge’ game was designed. To sustain engagement: ‘glucose challenges’ were offered to help participants focus on their BG ranges at certain times of the day. Outside of the group setting, remote monitoring of data could ‘flag*’ potential BG issues by e‐mail to enable timely intervention by the participant and/or facilitator.

## 4 INITIATING BEHAVIOUR CHANGE

As well as the novel content described above, the delivery style and interaction between facilitator and participant were considered key to supporting behaviour change. General guidance now suggests that empowerment and coaching models are best practice in long‐term condition management rather than education alone.^17^ Indeed, diabetes has been at the forefront of these developments and the role of the Diabetes Educator has been described as the ‘logical facilitator of change’.^18^

DAFNE educators are trained in patient empowerment and group learning,^19^ however, research identified the need for further training in other behaviour change approaches, such as Motivational Interviewing (MI), for example.^20^ Furthermore, Fredrix et al.'s^21^ research with DAFNE educators from Ireland revealed some hesitancy in delivering key behaviour change processes: goal setting and action planning. This was in terms of its perceived value within the curriculum, but also their confidence in facilitating these sessions.

Optimal action planning in diabetes may involve several smaller steps, a variety of strategies, be sufficiently flexible and acknowledge that life is not perfect.^18^ Within the patient empowerment framework that DAFNE already utilises, goals should be selected by the participant and feel meaningful and salient. Furthermore, supporting personal autonomy by healthcare professionals (HCPs) also has a positive association with HbA_1c_.^22^ Motivational interviewing (MI) lends itself particularly well to the goal‐setting process, as its purpose is to support patient autonomy, maximise rapport and minimise resistance to change and has been applied to the field of diabetes specifically.^23^

## 5 SUSTAINING BEHAVIOUR CHANGE

Setbacks are inevitable within a long‐term condition, and developing resilience is of increasing interest.^24^ Resilience is defined as coping with adversity when exposed to risk factors, such as living with a chronic disease. Attitudes to setbacks may also play a pivotal role in managing diabetes over the long term. Hilliard's diabetes resilience model^25^ suggests protective factors should be targeted within existing diabetes interventions, and this fits with the current revision of DAFNE. Protective factors include self‐compassion and HCP communication—both are associated with better glycaemic outcomes and buffer the impact of diabetes distress.^26^, ^27^ A ‘non‐judging’ stance is an example of how self‐compassion can be modelled by the Diabetes Educator.

Further advances in the field of action planning have also highlighted the role of ‘if … then?’ and ‘now …what?’ questions, so that participants can think about ways to cope if things do not go to plan or about future implications.^28^ This supports reflective and problem‐solving skills and also prevents goal‐setting from being ‘tokenistic’ or a one‐off activity that is not revisited.^21^ Within this context, Diabetes educators can provide a ‘coaching’ role using solution‐focused questions^29^ to encourage reflection, highlight pre‐existing skills/resources and uncover solutions.

In summary, DAFNE*plus* introduced new clinical, psychological and technological content and materials. It also targeted behaviour change processes more explicitly (such as action planning, problem solving and habit formation) and focused upon building long‐term resilience (e.g. self‐talk; reduce negative emotion, focus on past success). This paper outlines the training design and delivery for DAFNE*plus* facilitators.

## 6 TRAINING DEVELOPMENT AND METHODS

The training programme development was led by a diabetes clinical psychologist (NdZ) and collaboratively designed and delivered with two diabetes educators (CF and CG) and a behaviour change specialist (PC). To develop the training, NdZ audited two UK HCP training programmes: DAFNE^19^ and DESMOND^30^ (to establish current training practices) and reviewed HCP training recommendations from the literature. The trial collaborative working group also highlighted key targets for training based on previous evaluative research, current behavioural analyses and frontline clinical experience. The training programme was then developed, delivered and evaluated as part of a pilot study^11^ and then revised between iterations before being delivered in the trial centres.

A review of training literature around health psychology interventions recommends that training programmes should be standardised, attend to trainee differences, use role‐play and measure skill acquisition.^14^ Consequently, standardised DAFNE*plus* training materials, including the timetable, online modules, PowerPoint presentations and interactive activities (including role‐plays) were specifically written and manualised. This enabled consistency in delivery between sites and trainers.

As well as standardisation of materials and facilitator prior experience, consultation work with stakeholders and learning from the pilot highlighted the importance of engaging facilitators in the new training package who may have had doubts about revising DAFNE and/or limited capacity due to existing workloads.^31^ Consequently, didactic information was presented within online modules that could be done in the facilitators' own time (self‐directed learning), and live sessions were dedicated to interactive exercises, reflection and discussion. The training materials were designed to be engaging and meaningful by presenting diabetes/DAFNE*plus*‐specific examples, rather than general psychology principles. This material was brought to life using problem‐based learning^32^ (e.g. clinical vignettes) and real‐life data downloads and patient action plans. In addition to this, short video testimonials were played from DAFNE educators who had already piloted the programme and could speak about the added value they perceived.

In terms of developing facilitator confidence, a review of the literature highlighted the benefits of layered learning, role‐play (with corrective feedback) scripts and supervision.^33^ Consequently, teaching progressed from theory (via the online modules) to practice (via the live sessions), initially covering delivery of the group course, and then training for the individual follow‐up sessions once the first group course had been delivered. Role‐play learning was also layered with trainees observing trainer demonstrations first, before practicing in pairs, then small groups and then finally delivering to the whole group. Pre‐written scripts were available for the newer psychology sessions, and supervision was built into the training programme as outlined below.

## 7 TRAINING OVERVIEW

**FIGURE 1:**
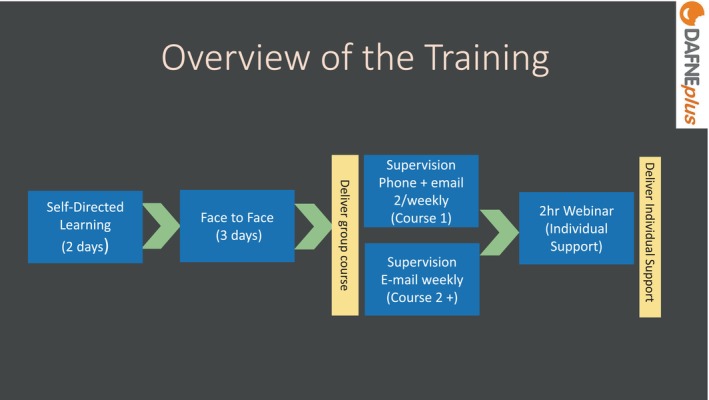
Overview of the training.

In total, 28 facilitators (trainees) were trained from six DAFNE centres. The training consisted of three parts: self‐directed learning modules (2 days), a live workshop (3 days) and a webinar (2 h) as illustrated in Figure 1.

Facilitators first completed three self‐directed learning modules that provided the background, theory and context for DAFNE*plus*. They then attended a 3‐day in‐person training workshop which provided interactive and experiential learning. During their first course delivery, facilitators received a support phone call before and e‐mail supervision after each week of the course. From the second course onwards, they received weekly e‐mail supervision. Additionally, after delivering their first course, facilitators attended a 2‐h webinar (with live Q&A) to train in the Individual Support (follow‐up) component of the programme.

The following section is divided into examples from the novel clinical, technological and psychological aspects of DAFNE*plus*, and what training methods and activities were used to support each area. The philosophy of the training team was to mirror the guidance given to participants during the group course ‘there's no such thing as failure, only feedback’. Consequently, there was a focus upon trying new things out (e.g. role plays) and the benefits of making mistakes in a supportive environment.

## 8 TRAINING CONTENT EXAMPLES

### 8.1 Clinical learning

In the self‐directed learning modules, all the clinical changes between standard DAFNE and DAFNE*plus* were described. This included simplified BG targets, simplified hypoglycaemia treatment advice, more emphasis on background insulin assessment and carb‐free meals in a day, carb counting in CPs or grams, aiming for 70% of BG readings within the target range and less emphasis on medical aspects of the annual review and more on positive long‐term health promotion.

**FIGURE 2:**
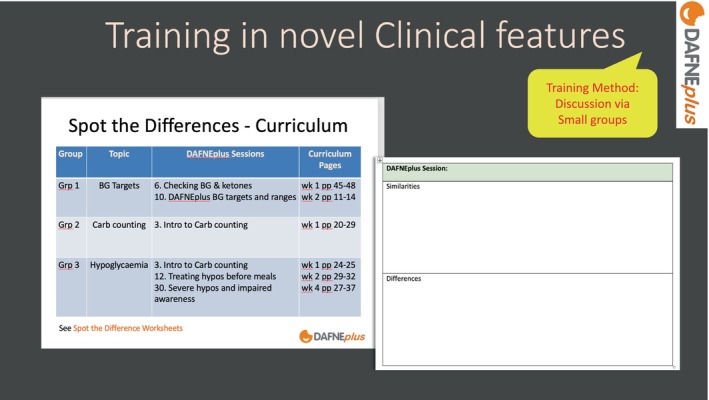
Training in novel clinical features.

In the live workshop, to bring these revisions to life and be more memorable, trainees were asked to look at specific sections of the curriculum and complete a ‘spot the differences’ worksheet within small groups. As well as changes in clinical guidance, trainees were directed to notice language changes, timetable changes and what previous content had been moved to e‐learning. Trainees then observed the Trainers (CG/CF) role‐play delivering the novel sessions/activities/tools based on these revisions and trainees were asked to make observation notes on what they had learnt (see Figure 2).

### 8.2 Technology learning

**FIGURE 3:**
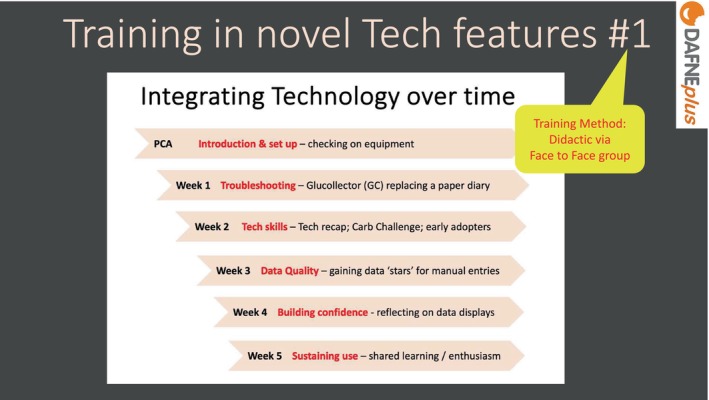
Training in novel technology features #1.

In the live workshop, trainees were taught to layer the learning regarding the new technology. Skills were broken down across the weeks, with a focus on mastering one area before moving on to the next as shown in Figure 3. This model also became a framework to return to in supervision to encourage pacing of the behavioural goals around technology.

**FIGURE 4:**
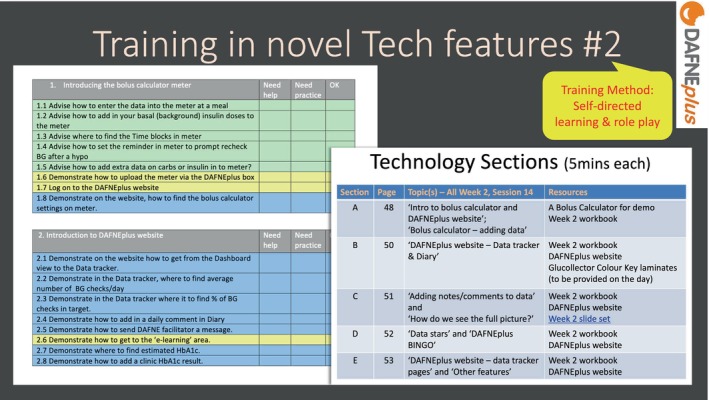
Training in novel technology features #2.

To increase familiarity with the new technology, including the website, trainees were provided with a checklist they could work through and assess their level of confidence (see Figure 4). This was part of the self‐directed learning. In the live workshop, this was developed further by role‐playing (in small groups) the technology‐related parts of the curriculum. For example, demonstrating the bolus calculator or showing features of the data tracker on the website.

To familiarise trainees with the more fun elements of the website, they were encouraged to play the ‘carb challenge’ game against each other in small teams. This was a novel feature of the website that involved looking at a photo of a meal and trying to guess the carb content. This game was repeated at the start of each training day to both increase familiarity with the website and encourage some light‐hearted interaction. This mirrored the delivery of the carb challenge game in the participant group course.

### 8.3 Psychology learning

**FIGURE 5:**
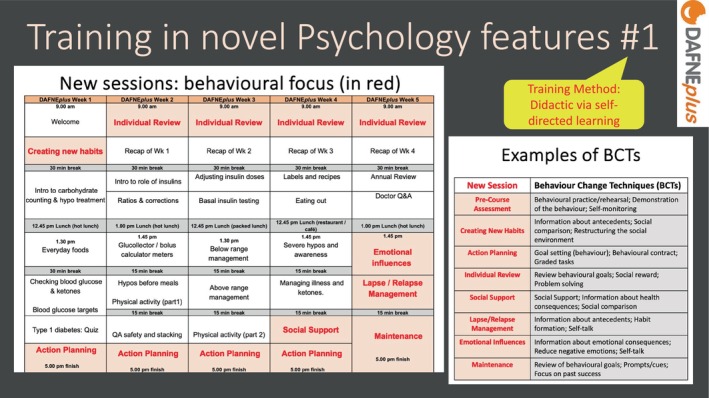
Training in novel psychology features #1.

In the self‐directed learning module, trainees were taught about the new behaviour change sessions and their place in the timetable, the theoretical model^34^ that supported their inclusion, and what specific behaviour change techniques were associated with each new or revised session† (see Figure 5).

**FIGURE 6:**
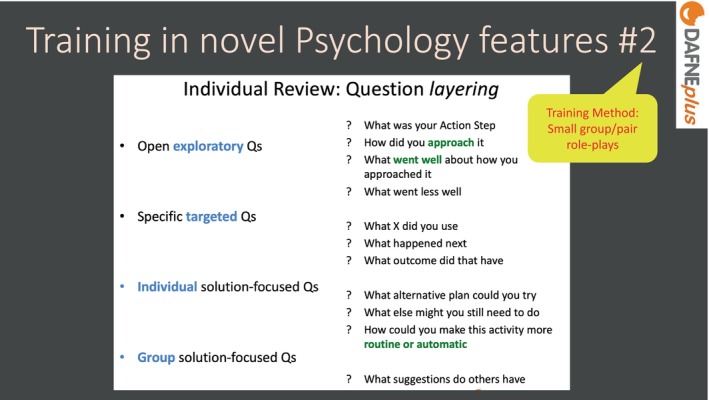
Training in novel psychology features #2.

In the live workshop, trainees were introduced to a question funnelling model, starting with open exploratory questions, moving to specific targeted questions and ending with solution‐focussed questions (see Figure 6). This coaching model could be used in the Individual Review sessions, when participants were reviewing their action plan from the week before. In small groups, trainees could role‐play this technique, using real‐life action plans from the pilot study.

Several resources were created for the new behaviour change sessions, such as visual metaphors (e.g. ‘the messy cupboard’), quote cards to make behaviour change principles more memorable (e.g. ‘no man is an island’—to highlight the role of social support) and visual aids depicting the vicious cycle that links thoughts, feelings and behaviours and how a lapse can turn into a relapse.‡ In the live workshop, the trainers (PC/CG/CF) would demonstrate how to run these sessions using these materials. Each trainee would then be allocated a session to practice and then role‐play with the whole group. This was observed by the trainers who could provide feedback.

### 8.4 Individual Support (follow‐up) sessions

**FIGURE 7:**
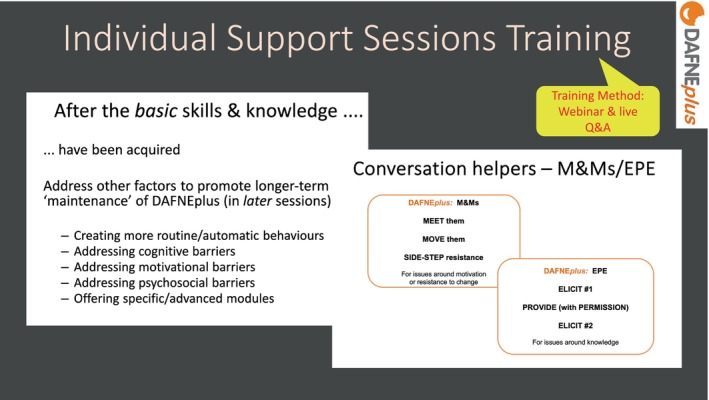
Individual support sessions training.

To support trainees to deliver the Individual Support sessions, a 2‐h webinar (+ live Q&A) was provided. This included didactic teaching on how to tailor the course material to the participant's life circumstances, to address additional barriers to putting DAFNE*plus* learning into action (see Figure 7) and how to use the appointment preparation forms§ to be primed for a more effective consultation. In the live workshop, trainees were introduced to some ‘conversation helpers’ based on motivational interviewing principles (e.g. elicit–provide–elicit) that could be used to manage resistance to change.

## 9 TRIAL SUPERVISION AND SUPPORT

**FIGURE 8:**
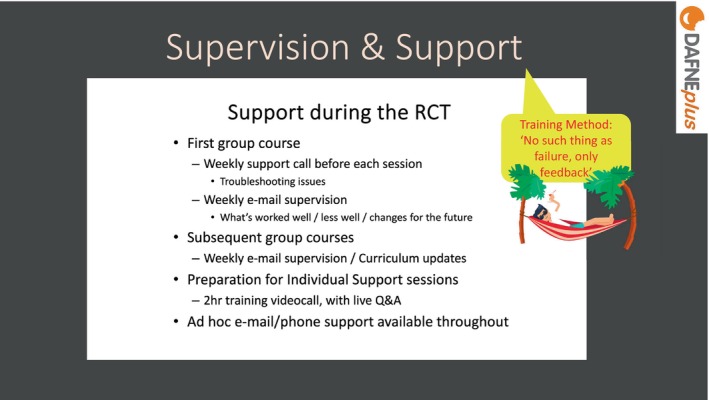
Supervision and support.

Facilitators were offered regular supervision as shown in Figure 8. As facilitators did not have an opportunity to observe a course or run a course before the trial started, they were offered more support for their first course, in the form of an additional weekly telephone call before each session. Subsequent courses had weekly e‐mail supervision, and ad hoc support was also available from the training team.

## 10 DISCUSSION

This paper has described the training provided to the facilitators who delivered the DAFNE*plus* intervention. It outlines the research background, theory and consultation that informed the training's content, format and delivery. The training comprised self‐directed learning, observation, discussion and role‐play. The learning was layered to introduce increasing levels of complexity in both knowledge and skills. This paper has presented examples of the training content for the novel clinical, technological and psychological components from both the group course and individual support sessions. Training was consolidated by the provision of scripts (for novel sessions) and regular supervision.

In terms of how the training was received, Lawton et al. conducted interviews with DAFNE*plus* facilitators exploring their training and delivery experience.^35^ These interviews reported high levels of ‘buy‐in’ once initial concerns about delivering more psychology content had been addressed. The interviews highlighted that the bespoke training (including use of role‐play) and supervision (including one‐to one feedback) were pivotal in supporting the transformation from educator to facilitator. The use of scripts also promoted familiarity with and confidence in delivering novel sessions and coaching approaches. For many, the transformation was so powerfully felt that they reported DAFNE*plus* practices and principles (intentionally and/or inadvertently) creeping into their everyday clinical practice, such as setting smaller, behaviour‐focused goals (not just target focused) and actively commenting upon signs of progress (not just issues of concern) in their consultations. Facilitators were keen for these ideas to become more mainstream and commented that a system/team‐wide approach would need to support this.

The above changes partly resonate with those of other studies which have explored diabetes HCPs' experiences of adopting a more psychological role. For example, the process of transformation, and the enthusiasm to use ideas outside of the trial context, fits with Findlay‐White's report^36^ of ‘reinvigorated professionals’ from an interview study with HCPs who had attended a diabetes counselling and empowerment course. In addition, facilitators' caution about wider service changes, being required to facilitate a more psychologically informed approach, is mirrored in Graves et al.'s^37^ study with primary care nurses trained in psychological approaches for diabetes, who felt the lack of department support was a barrier to implementation. However, previous concerns of over‐stepping boundaries^37^; the need to ‘fix’ patients^38^; and discomfort of delivering the ‘touchy‐feely’ sessions^21^ were not found in Lawton et al.'s^35^ study, suggesting that the DAFNE*plus* training may have enabled HCPs to have more confidence in adopting and delivering the programme's psychologically informed content. Our training protocol also contrasts with the fidelity evaluation of the English Diabetes Prevention Programme^39^ which found that not all staff were trained in key self‐regulatory BCTs, such as action planning, or experienced demonstrations and role‐play as part of their training.

In terms of limitations of the training programme, unlike standard DAFNE training, the facilitators did not have a chance to observe a group before delivering their own. Due to resource constraints, a formal pre‐/post‐training competency assessment in confidence/competence was not performed. In addition, the timing of the Covid pandemic meant that some facilitators had a lengthy gap between running courses. To address some of these constraints, extra supervision was provided for the first run of the course, with a weekly pre‐session phone call and post‐session e‐mail, and a booster training session after the lockdown period. In terms of observation, trainers demonstrated all the new sessions (although in a role‐play context) and facilitators received informal feedback on their own skills during group role‐play sessions.

Future publications will report the final trial results for DAFNE*plus* v standard DAFNE and consequently whether the training helped to deliver an intervention that was effective. Further reports will also include a fidelity analysis (both self‐report and objective measurement) which will help establish whether facilitators delivered the programme as intended. Future developments may include shorter, e‐learning training modules for the specific elements of the curriculum that trial facilitators are already transferring to routine care.^35^ In addition, the design of simple and effective measures to assess skills post‐training and beyond, for example, DOT (DESMOND Observational Tool),^40^ may ensure facilitators continue to develop their skills and confidence.

In conclusion, DAFNE*plus* provided a 5‐day training course that enabled facilitators to deliver the DAFNE*plus* programme. The aim of the training was to explain the revised elements of the curriculum, support with new technology components and increase confidence in initiating and sustaining behaviour change. Evaluation to date^35^ suggests that facilitators felt the training had been beneficial, credible and provided benefits that had extended beyond the trial context. Further work on fidelity, facilitator competency and the development of accessible training modules will support the ongoing training needs for diabetes HCPs.

## CONFLICT OF INTEREST STATEMENT

Simon Heller undertakes consultancy with Eli Lilly, NovoNordisk, Zealand Pharma, Vertex, Zucara, Medtronic and receives research support from Dexcom Inc. None of the other authors has any conflicts of interest.

## Supporting information


Data S1.

## Footnotes

* The software was programmed to spot unhelpful patterns of behaviour, from downloads, which might increase the likelihood of hyper/hypoglycaemic events and to e‐mail an alert (flag) to the participant in the first instance.

^†^
Further detail on how relevant BCTs were translated into DAFNEplus‐specific materials can be found in Stanton‐Fay et al. (Table 3)^9^.

^‡^
See Data S1 for examples of these visual aids.

^§^
Forms sent out *before* the appointment with structured prompts to help both participant and facilitator reflect on downloaded data, behavioural patterns and priorities to be discussed at the next appointment.

## References

[1] Heller S , Lawton J , Amiel S , et al. Improving Management of Type 1 Diabetes in the UK: the Dose Adjustment for Normal Eating (DAFNE) Programme as a Research Test‐Bed. A Mixed‐Method Analysis of the Barriers to and Facilitators of Successful Diabetes Self‐Management, a Health Economic Analysis, a Cluster Randomised Controlled Trial of Different Models of Delivery of an Educational Intervention and the Potential of Insulin Pumps and Additional Educator Input to Improve Outcomes. NIHR Journals Library; 2014:1‐188. 25642502

[2] DAFNE*plus* Group Component Logic Model. 2024. https://www.sheffield.ac.uk/media/43569/download. Accessed January 21, 2025.

[3] DAFNE*plus* Individual Component Logic Model. 2024. https://www.sheffield.ac.uk/media/45456/download. Accessed January 21, 2025.

[4] DAFNEplus Glucollector Logic Model (2024). https://www.sheffield.ac.uk/media/41996/download. Accessed January 21, 2025.

[5] Eissa MR , Good T , Elliott J , Benaissa M . Intelligent data‐driven model for diabetes diurnal patterns analysis. IEEE J Biomed Health Inform. 2020;24(10):2984‐2992. doi:10.1109/JBHI.2020.2975927 32092021

[6] Lawton J , Rankin D , Cooke D , et al. Patients' experiences of adjusting insulin doses when implementing flexible intensive insulin therapy: a longitudinal, qualitative investigation. Diabetes Res Clin Pract. 2012;98(2):236‐242. doi:10.1016/j.diabres.2012.09.024 23084281

[7] Rankin D , Cooke DD , Elliott J , Heller SR , Lawton J , UK NIHR DAFNE Study Group . Supporting self‐management after attending a structured education programme: a qualitative longitudinal investigation of type 1 diabetes patients' experiences and views. BMC Public Health. 2012;12(652):652. doi:10.1186/1471-2458-12-652 22891794 PMC3490905

[8] Rankin D , Cooke DD , Clark M , et al. How and why do patients with type 1 diabetes sustain their use of flexible intensive insulin therapy? A qualitative longitudinal investigation of patients' self‐management practices following attendance at a dose adjustment for Normal eating (DAFNE) course. Diabet Med. 2011;28(5):532‐538. doi:10.1111/j.1464-5491.2011.03243.x 21244477

[9] Stanton‐Fay SH , Hamilton K , Chadwick PM , et al. The DAFNE*plus* programme for sustained type 1 diabetes self management: intervention development using the behaviour change wheel. Diabet Med. 2021;38(5):e14548. doi:10.1111/dme.14548 33617669

[10] Hamilton K , Stanton‐Fay SH , Chadwick PM , et al. Sustained type 1 diabetes self‐management: specifying the behaviours involved and their influences. Diabet Med. 2021;38(5):e14430. doi:10.1111/dme.14430 33073393 PMC8247296

[11] Breckenridge JP , Gossage‐Worrall R , Chadwick P , et al. The collaborative working group method for pre‐trial knowledge mobilisation: a qualitative evaluation of a structured process for iteratively refining a complex intervention (DAFNE*plus*). Pilot Feasibility Stud. 2024;10(1):154. doi:10.1186/s40814-024-01576-3 39709454 PMC11662412

[12] DAFNE*plus* Programme Theory. 2024. https://www.sheffield.ac.uk/media/41995/download. Accessed January 21, 2025

[13] Coates E , Amiel S , Baird W , et al. Protocol for a cluster randomised controlled trial of the DAFNE*plus* (dose adjustment for Normal eating) intervention compared with 5×1 DAFNE: a lifelong approach to promote effective self‐management in adults with type 1 diabetes. BMJ Open. 2021;11(1):e040438. doi:10.1136/bmjopen-2020-040438 PMC7813353 33462097

[14] Bellg AJ , Borrelli B , Resnick B , et al. Enhancing treatment fidelity in health behavior change studies: best practices and recommendations from the NIH behavior change consortium. Health Psychol. 2004;23(5):443‐451. doi:10.1037/0278-6133.23.5.443 15367063

[15] NHS England Language Matters: language and diabetes. 2023. https://www.england.nhs.uk/long‐read/language‐matters‐language‐and‐diabetes/. Accessed January 20, 2025.

[16] Engel L , Cummins R . Impact of dose adjustment for normal eating in Australia (OzDAFNE) on subjective wellbeing, coping resources and negative affects in adults with type 1 diabetes: a prospective comparison study. Diabetes Res Clin Pract. 2011;91(3):271‐279. doi:10.1016/j.diabres.2010.11.023 21146889

[17] Bodenheimer T , MacGregor K , Sharifi C . Helping patients manage their chronic conditions. California HealthCare Foundation; 2005.

[18] Burke SD , Sherr D , Lipman RD . Partnering with diabetes educators to improve patient outcomes. Diabetes Metab Syndr Obes Targets Ther. 2014;7:45‐53. doi:10.2147/dmso.s40036 PMC3926770 24550679

[19] Oliver L , Thompson G . The DAFNE collaborative. Experiences of developing a nationally delivered evidence‐based, quality‐assured programme for people with type 1 diabetes. Pract Diab Int. 2009;26:371‐377. doi:10.1002/pdi.1424

[20] Byrne JL , Davies MJ , Willaing I , et al. Deficiencies in postgraduate training for healthcare professionals who provide diabetes education and support: results from the diabetes attitudes, wishes and needs (DAWN2) study. Diabet Med. 2017;34(8):1074‐1083. doi:10.1111/dme.13334 28195662

[21] Fredrix M , Byrne M , Dinneen S , McSharry J . ‘It's an important part, but I am not quite sure that it is working’: educators' perspectives on the implementation of goal‐setting within the ‘DAFNE’ diabetes structured education programme. Diabet Med. 2019;36(1):80‐87. doi:10.1111/dme.13813 30175873

[22] Williams GC , Freedman ZR , Deci EL . Supporting autonomy to motivate patients with diabetes for glucose control. Diabetes Care. 1998;21(10):1644‐1651. doi:10.2337/diacare.21.10.1644 9773724

[23] Steinberg MP , Miller WR . Motivational interviewing in diabetes care. Guilford Publications; 2015.

[24] Skedgell KK , Cao VT , Gallagher KA , Anderson BJ , Hilliard ME . Defining features of diabetes resilience in emerging adults with type 1 diabetes. Pediatr Diabetes. 2021;22(2):345‐353. doi:10.1111/pedi.13136 33034097 PMC12168199

[25] Hilliard ME , Harris MA , Weissberg‐Benchell J . Diabetes resilience: a model of risk and protection in type 1 diabetes. Curr Diab Rep. 2012;12(6):739‐748. doi:10.1007/s11892-012-0314-3 22956459

[26] Akbari M , Seydavi M , Rowhani NS , Nouri N . Psychological predictors of treatment adherence among patients with diabetes (types I and II): modified information‐motivation‐behavioural skills model. Clin Psychol Psychother. 2022;29(6):1854‐1866. doi:10.1002/cpp.2746 35510374

[27] Sandham C , Deacon E . The role of self‐compassion in diabetes management: a rapid review. Front Psychol. 2023;14:1123157. doi:10.3389/fpsyg.2023.1123157 37063529 PMC10098353

[28] Mann T , de Ridder D , Fujita K . Self‐regulation of health behavior: social psychological approaches to goal setting and goal striving. Health Psychol. 2013;32(5):487‐498. doi:10.1037/a0028533 23646832

[29] Davis ED , Vander Meer JM , Yarborough PC , Roth SB . Using solution‐focused therapy strategies in empowerment‐based education. Diabetes Educ. 1999;25(2):249‐252. doi:10.1177/014572179902500210 10531850

[30] Davies MJ , Heller S , Skinner TC , et al. Diabetes education and self Management for Ongoing and Newly Diagnosed Collaborative. Effectiveness of the diabetes education and self management for ongoing and newly diagnosed (DESMOND) programme for people with newly diagnosed type 2 diabetes: cluster randomised controlled trial. BMJ. 2008;336(7642):491‐495. doi:10.1136/bmj.39474.922025.BE 18276664 PMC2258400

[31] Lawler J , Leary A , Walden E , Stanisstreet D , Punshon G . The workload of the diabetes specialist nurse workforce in the UK. J Diabetes Nurs. 2020;23(6):12589.

[32] Tschannen D , Aebersold M , Sauter C , Funnell MM . Improving nurses' perceptions of competency in diabetes self‐management education through the use of simulation and problem‐based learning. J Contin Educ Nurs. 2013;44(6):257‐263. doi:10.3928/00220124-20130402-16 23565600

[33] Masava B , Nyoni CN , Botma Y . Scaffolding in health sciences education Programmes: an integrative review med. Sci Educ. 2022;255:8420. 10.1007/s40670-022-01691-x PMC10060462 37008420

[34] Michie S , van Stralen MM , West R . The behaviour change wheel: a new method for characterising and designing behaviour change interventions. Implement Sci. 2011;6:42. doi:10.1186/1748-5908-6-42 21513547 PMC3096582

[35] Lawton J , Rankin D , Scott E , et al. From educator to facilitator: healthcare professionals' experiences of, and views about, delivering a type 1 diabetes structured education programme (DAFNE*plus*) informed by behavioural science. Diabet Med. 2024;41(8):15375. doi:10.1111/dme.15375 38837475

[36] Findlay‐White F , Dornan T , Davies M , Archer A , Kilvert A , Fox C . From fixer to facilitator: an interpretative phenomenological study of diabetes person‐centred counselling and empowerment‐based education. F1000Res. 2023;11(78):78. doi:10.12688/f1000research.73596.2 38434003 PMC10904953

[37] Graves H , Garrett C , Amiel SA , Ismail K , Winkley K . Psychological skills training to support diabetes self‐management: qualitative assessment of nurses' experiences. Prim Care Diabetes. 2016;10(5):376‐382. doi:10.1016/j.pcd.2016.03.001 27006306

[38] Pill R , Rees ME , Stott NC , Rollnick SR . Can nurses learn to let go? Issues arising from an intervention designed to improve patients' involvement in their own care. J Adv Nurs. 1999;29(6):1492‐1499. doi:10.1046/j.1365-2648.1999.01037.x 10354245

[39] Hawkes RE , Cameron E , Miles LM , French DP . The Fidelity of training in behaviour change techniques to intervention Design in a National Diabetes Prevention Programme. Int J Behav Med. 2021;28(6):671‐682. doi:10.1007/s12529-021-09961-5 33559009 PMC8551141

[40] Cradock S , Daly H , Bonar D , Carey ME , Cullen M , Doherty Y . Charting excellence; developing effective methods for quality assuring educators as part of the DESMOND Programme. Diabet Med. 2008;25(Suppl 1):18.

