# Supplementary material for: Training DAFNE plus facilitators in novel behaviour change approaches: A template for training design and delivery
Source: Diabet Med. 2025 Jun 22;42(9):e70078. doi: 10.1111/dme.70078 (PMC12352730; doi:10.1111/dme.70078)

**Supplementary Material**

**TRAINING CONTENT EXAMPLES**

*Psychology learning (3^rd^ paragraph)*

Several resources were created for the new behaviour change sessions, such as visual metaphors (e.g. ‘the messy cupboard’), quote cards to make behaviour change principles more memorable (e.g. ‘no man is an island’ – to highlight the role of social support) and visual aids depicting the vicious cycle that links thoughts, feelings and behaviours, and how a lapse can turn into a relapse. In the live workshop, the trainers (PC/CG/CF) would demonstrate how to run these sessions using these materials. Each trainee would then be allocated a session to practice and then role-play with the whole group. This was observed by the trainers who could provide feedback.

**Examples of visual aids in DAFNEplus**

**Visual metaphor: ‘The Messy Cupboard’**

To help participants understand that attending a DAFNEplus course might feel overwhelming before it starts to feel better and more manageable.


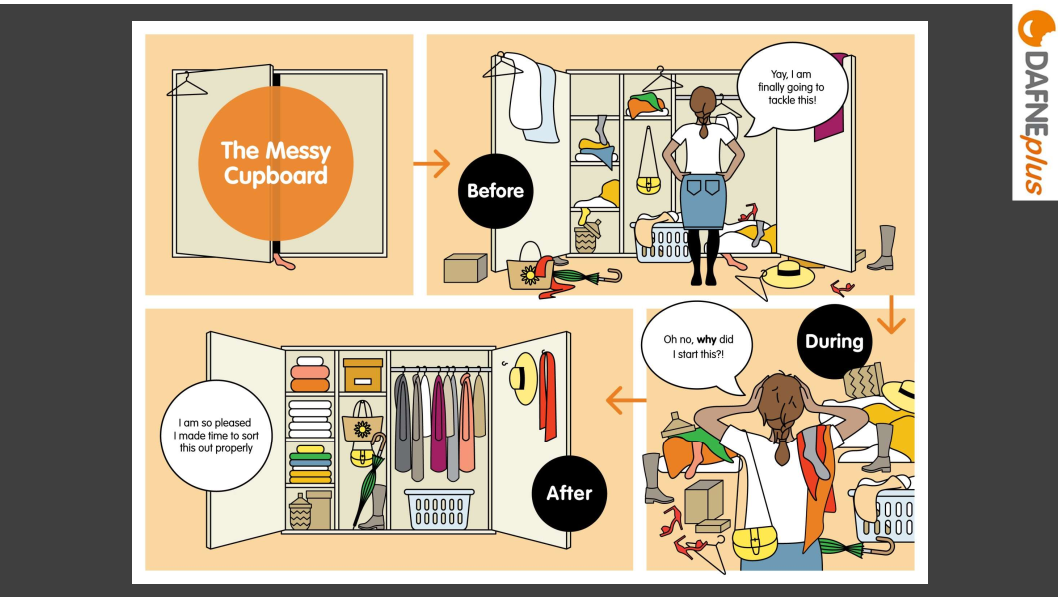


**Quote card on social support: ‘No man or woman is an island’**

To help participants appreciate that it’s OK to ask for help/involve other people and to consider different ways to do this within the ‘social support’ session.


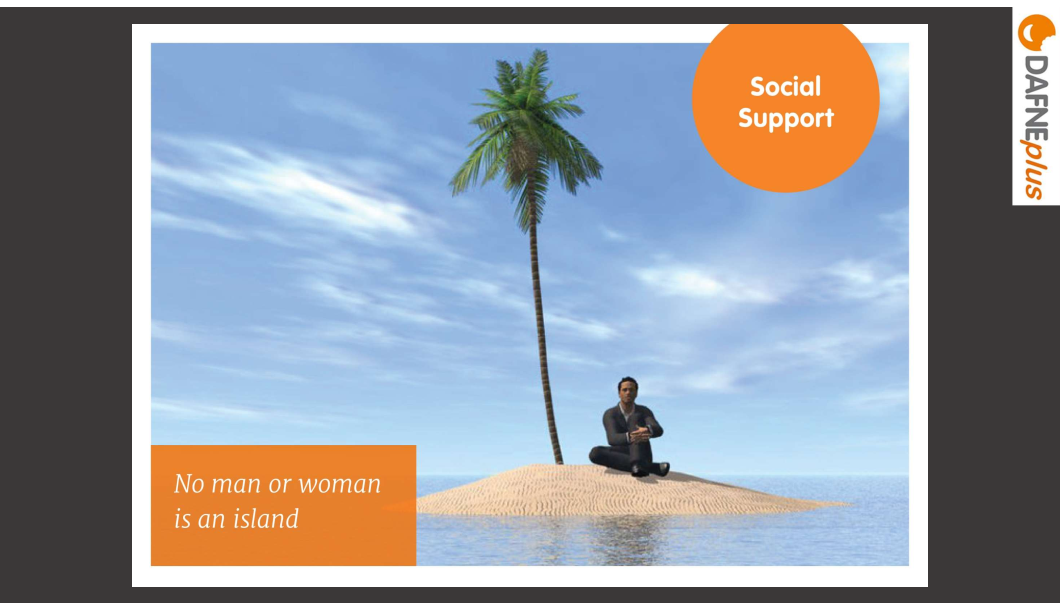


**Vicious cycle: ‘How a lapse turns into a relapse’**

To highlight how thoughts, feelings and behaviours can turn a temporary setback into a longer-term problem.


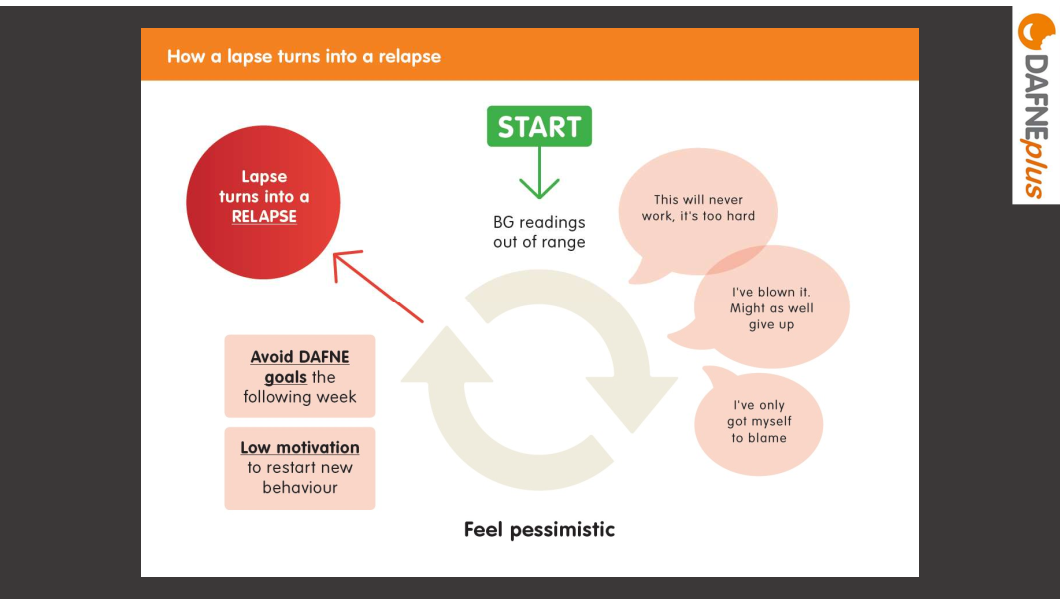

Supplement: Supplementary file 1 — Data S1. [file DME-42-e70078-s001.docx]
